# Genistein Reduces the Risk of Local Mammary Cancer Recurrence and Ameliorates Alterations in the Gut Microbiota in the Offspring of Obese Dams

**DOI:** 10.3390/nu13010201

**Published:** 2021-01-11

**Authors:** Fabia de Oliveira Andrade, Fang Liu, Xiyuan Zhang, Mariana Papaleo Rosim, Caroline Dani, Idalia Cruz, Thomas T. Y. Wang, William Helferich, Robert W. Li, Leena Hilakivi-Clarke

**Affiliations:** 1 Department of Oncology, Georgetown University, Washington, DC 20057, USA; deoli038@umn.edu (F.d.O.A.); xiyuan.zhang@nih.gov (X.Z.); mariana.rosim@gmail.com (M.P.R.); carolinedani@yahoo.com.br (C.D.); cruzi@georgetown.edu (I.C.); 2 College of Food Science and Engineering, Ocean University of China, Qingdao 266555, China; fliu19910205@gmail.com; 3 United States Department of Agriculture, Beltsville Human Nutrition Center, Diet, Genomics and Immunology Laboratory, Beltsville, MD 20705, USA; tom.wang@usda.gov; 4 Department of Food Science and Human Nutrition, University of Illinois Urbana-Champaign, Urbana, IL 1801, USA; helferic@illinois.edu; 5 United States Department of Agriculture, Agricultural Research Service, Animal Genomics and Improvement Laboratory, Beltsville, MD 20705, USA; robert.li@usda.gov

**Keywords:** breast cancer, tamoxifen therapy, antiestrogen resistance, genistein, gut microbiota, tumor immune genes

## Abstract

The risk of recurrence of estrogen receptor-positive breast cancer remains constant, even 20 years after diagnosis. Recurrence may be more likely in patients pre-programmed for it already in the womb, such as in the daughters born to obese mothers. Maternal obesity persistently alters offspring’s gut microbiota and impairs tumor immune responses. To investigate if the gut dysbiosis is linked to increased risk of mammary cancer recurrence in the offspring of obese rat dams, we fed adult offspring genistein which is known to have beneficial effects on the gut bacteria. However, the effects of genistein on breast cancer remain controversial. We found that genistein intake after tamoxifen response prevented the increased risk of local recurrence in the offspring of obese dams but had no effect on the control offspring. A significant increase in the abundance of inflammatory *Prevotellaceae* and *Enterobacteriaceae*, and a reduction in short-chain fatty acid producing *Clostridiaceae* was observed in the offspring of obese dams. Genistein supplementation reversed these changes as well as reversed increased gut metabolite N-acetylvaline levels which are linked to increased all-cause mortality. Genistein supplementation also reduced genotoxic tyramine levels, increased metabolites improving pro-resolving phase of inflammation, and reversed the elevated tumor mRNA expression of multiple immunosuppressive genes in the offspring of obese dams. If translatable to breast cancer patients, attempts to prevent breast cancer recurrences might need to focus on dietary modifications which beneficially modify the gut microbiota.

## 1. Introduction

Approximately half of all infants in the USA today have been born to an overweight or obese mother [1], and this can have significant adverse effects on their later health [2], including increasing breast cancer risk and mortality. Exposure to maternal obesity in the womb, leading to a high birth weight (>4000 g/8.8 lb), increases a daughter’s risk of developing breast cancer and breast cancer mortality in humans [3,4,5] and in animal models [6,7]. We found that rat offspring born to dams fed an obesity-inducing high fat diet (HFD) exhibited a highly significant increase in local mammary tumor recurrence after initially responding to tamoxifen therapy [7]. Thus, interventions to prevent breast cancer mortality in the offspring of obese mothers should focus on the period starting after endocrine therapy has been completed.

Patients with lymph node-negative, estrogen receptor-positive (ER+) breast cancers show excellent 90–95% five-year survival [8]. However, after five years of endocrine therapy ER+ breast cancer survivors remain at a constant risk of developing recurrence [9]. Consequently, 15 years after completion endocrine therapy almost every fourth patient with early stage, local ER+ disease had recurred and 15% had died of breast cancer [10]. At the same time frame, 52% of those survivors who had locally advanced ER+ breast cancer at diagnosis had recurred and 49% died from breast cancer [10]. Although continuing endocrine therapies for additional five years reduces the risk of recurrence [8], endocrine therapies are linked to serious side effects, such as tamoxifen increasing the risk of endometrial cancer and blood clots, and aromatase inhibitors causing bone loss and joint and muscle pain [11]. A more attractive option to a continuous endocrine therapy is to identify safe post-therapy strategies to prevent breast cancer recurrences.

Maternal obesity permanently affects many biological pathways in the offspring, including the gut microbiota. The genes and gene products of the microbiota form the microbiome, which impact a wide range of targets such as the host’s immune system [12]. The gut microbiota is first established early in life [13], possibly already in utero. It undergoes changes during early postnatal and childhood development [14], and then remains relatively stable in adulthood [15]. Several studies have shown that maternal HFD and excessive pregnancy weight gain are associated with a gut dysbiosis in the offspring in humans [16,17,18], primates [19] and mice [20,21,22]. Gut dysbiosis is defined here as any perturbation of the normal microbiome content resulting a disruption in the symbiotic relations between the host and host’s microbes. The main reason why it is not feasible to define gut dysbiosis as an increased abundance of specific microbes and reduced abundance of others is that the diversity among the microbiomes of two different individuals is confounding, especially when compared with their human genomic variation. Human genomes are about 99.9% identical to each other [23], whilst their gut microbiome typically is only 10–20% similar [24]. Thus, each individual has a unique gut microbiota [25].

Genistein, a compound found in soy foods, has several biological properties that can result a reversal of the high rate of mammary tumor recurrence in the HFD offspring. Genistein activates anti-tumor immune responses [26], inhibits immunosuppression [27], and suppresses inflammatory factors [28]. In addition, genistein might reverse gut dysbiosis [29]. In an earlier study, when genistein was given to pregnant mouse dams also fed HFD, it increased the abundance of multiple short-chain fatty acid (SCFA) producing bacteria, including *Akkermansia* and *Clostridium* in the male offspring [30]. SCFAs have been linked to several anti-cancer mechanisms, including being anti-inflammatory [31]. Since the majority of children born to obese mothers have not been exposed to genistein in the womb, we investigated here if adding genistein to a diet of adult HFD offspring reverses their gut dysbiosis. In addition, we determined if the adult genistein intake can reverse gut dysbiosis and suppression of anti-tumor immunity in the offspring born to HFD fed dams. In humans, maternal obesity reduces monocyte and dendritic cell ex-vivo response and CD4+ T helper cell numbers and increases plasma levels of interferon α (IFNα) and interleukin 6 (IL6) in the umbilical cord blood, compared with offspring born to lean mothers [32]. We found earlier that maternal HFD impairs antigen presentation and anti-tumor CD8+ T cell activation in the offspring’s tumor microenvironment (TME) [7]. Since the composition of the gut microbiota can drive inflammatory and immune responses, it is possible that the increased inflammation and impaired anti-tumor immunity in the offspring of obese mothers is linked to their gut dysbiosis.

Our results in this study indicated that genistein reversed the increased abundance of inflammatory *Enterobacteriaceae* [33] and upregulated the reduced abundance of anti-inflammatory, SCFA-producing *Clostridium* [34] in the HFD offspring. Genistein also inhibited genotoxic bacterial metabolite tyramine as well as upregulated metabolites involved in the pro-resolving phase of inflammation in the offspring of rats fed HFD during pregnancy. Further, genistein upregulated the expression of anti-tumor immune response genes in the TME. Importantly, supplementing HFD offspring with genistein after tamoxifen response reversed their increased risk of local mammary tumor recurrence.

## 2. Materials and Methods

Maternal dietary exposures: Female Sprague Dawley rats at 3 weeks old were purchased from Charles River and fed an AIN93G-based HFD diet or control diet for 6 weeks before mating and throughout pregnancy. The HFD diet contained 50% of Kcal from fat (81% Crisco, 19% corn oil) and the control diet contained 13% of Kcal from fat (50% Crisco, 50% corn oil) [7]. After offspring were born, they were only fed modified AIN93G control diet.

Mammary tumor model: ER+ mammary tumors were induced on 50-day-old female offspring of dams fed control or HFD during pregnancy by 10 mg of 7,12-dimethylbenz[a]anthracene (DMBA) in 1 mL peanut oil, administered orally.

Tamoxifen and genistein treatments: Genistein supplementation started when tamoxifen treatment started. When at least one mammary tumor developed and the tumor reached a size of 13 mm in diameter, control and HFD offspring were divided to two additional groups: those receiving 340 ppm tamoxifen citrate in an AIN93G control diet, or tamoxifen and 500 ppm genistein. Tumor response was monitored by palpation and measured weekly with a caliper (length × width) and defined as complete response when a tumor disappeared and was gone for at least 6 weeks, partial response when a tumor stopped growing or shrunk, de novo resistance when a tumor never responded and kept growing and acquired resistance when tumor appeared and kept growing during tamoxifen treatment.

Genistein supplementation started after tamoxifen response: When at least one tumor per rat reached 11 mm in diameter, a rat started to receive 340 ppm tamoxifen citrate via diet. Tumor response was monitored as stated before, and rats from control and HFD groups with completely or partially responding tumors were divided in two other groups that received tamoxifen only or tamoxifen with 500 ppm genistein by diet for more 9 weeks. After tamoxifen was removed, rats on genistein continued consuming it for 10 weeks during which recurrences were monitored. Tumors were considered recurrent when it grew back into the same location where it was initially when TAM therapy started and when it reached again a size of 11mm in diameter.

Experimental conditions and tissue collection: Rats were maintained on a 12-h light-dark cycle at 22 °C with free access to diet and water. Rats were euthanized when the tumor burden reached 10% of body weight or at the end of the tumor monitoring period. Mammary tumors and feces were collected from each rat at euthanasia, transferred to liquid nitrogen and maintained at −80 °C.

Assessing changes in immune response markers in tumor microenvironment: RNA was extracted from mammary tumors with RNeasy Mini kit (Qiagen, Germantown, MD, USA) according to the manufacturer protocol. RNA was converted to cDNA using a High-Capacity cDNA reverse transcription kit (Applied Biosystems, Foster City, CA, USA) in a PTC-100 thermal cycler (Bio-Rad, Hercules, CA, USA). qRT-PCR was performed using BrightGreen qPCR Master Mix-ROX (abm, Richmond, BC, Canada) in QuantStudio 12K Flex Real-Time PCR System. mRNA levels for target genes were quantified using a Relative Standard Curve method normalized by housekeeping gene *Hprt1* for rat tissue. Primers for *Cd8*, *Foxp3*, *Tgfβ1*, *Pd1*, *Pdl1*, *Ctla4*, *Il6*, and *Hprt1* were designed using IDT tool primer design (Integrated DNA Technologies, IA, Table S1)

Statistical analysis for tumor response and gene expression: Tumor response to tamoxifen and tumor recurrence was assessed by a Chi-square test. Parametric t test, or two-way ANOVA followed by Holm-Sidak post-hoc test was used to assess differences in gene expression between two or more groups, respectively. When the data did not pass the normality and equal variance test, a non-parametric test was used. Statistical analysis was performed using Sigma Plot 11.0 and graphs for tumor response, recurrence and gene expression were designed using GraphPad Prism 8. The differences were considered statistically significant when p values were equal to or less than 0.05.

16S rRNA gene sequencing: A microbiome standard with a defined composition was obtained from Zymo Research (Irvine, CA, USA). The microbial community standard consists of three Gram^-^ and five Gram^+^ bacteria as well as two yeasts with a defined composition. In addition, nuclease free water was used as a non-DNA template (negative) control. Both negative controls and the microbiome standard were processed along with experimental samples following the same protocol and parameters to validate the entire workflow, from total DNA extraction and sequencing to data analysis.

Total DNA was extracted from colon contents as previously described [35]. Briefly, a QIAamp Fast DNA Stool Mini Kit (Qiagen, Germantown, MD, USA) was used with some modifications. First, a bead-beating bacterial cell wall disruption procedure using a FastPrep 5G instrument and Lysing Matrix E (MP Biomedicals, Irvine, CA, USA) was added. Second, lysis at 70 °C was extended to 8 min.

The hypervariable V3–V4 regions of the 16S rRNA gene amplification and sequencing were performed [36]. The primer sequences were as follows: forward primer, 341/357F, CCTACGGGNGGCWGCAG; reverse primer, 805/785R: GACTACHVGGGTATCTAATCC. A total of 20 cycles of PCR amplification was conducted. The amplified products from individual samples were purified using Agencourt AMPure XP beads (Beckman Coulter, Danvers, MA, USA). The purified PCR products were quantified using BioAnalyzer 2100 DNA 7500 chips and pooled based on an equal molar ratio and their respective samples-specific barcodes. The pooled libraries were sequenced using an Illumina MiSeq Reagent Kit v3 (2 × 255 cycles) as described previously [37,38].

Bioinformatic analysis of 16S rRNA gene sequences: Feature or OTU tables were generated using the Quantitative Insights into Microbial Ecology QIIME1 (v.1.9.1) [38] and QIIME2 (v 2019.07) pipelines [39].

The quality of raw reads was checked using FastQC v0.11.2. The sequences with low quality score and the four maximally degenerate bases (NNNN) at the most 5′end of the primer were removed using Trimmomatic v0.38. The paired end reads were merged using join_paired_ends.py with the following parameter settings: the minimum overlap length was 20 bp and the maximum allowed mismatches within the overlapping region was 5%. The command pick_closed_reference_otus.py was used for OTU picking. Taxonomy assignment was based on the Greengenes database (v13.8). *Alpha_diversity.py* command line was used for alpha-diversity index extraction at an OTU level. Several tools, such as NMDS, principal coordinate analysis (PCoA), PERMANOVA, and ANOSIM, were used for beta-diversity analysis based on various distance matrices. PICRUSt (v1.1.2) was used to predict metagenome functional contents from 16S rRNA marker gene survey data with default parameters based on the OTU table generated using the closed-reference protocol [40]. QIIME2: all reads were pooled using the *qiime tools import* script. The *qiime dada2 denoise-paired* was used for sequence quality control. The subsequent procedures were conducted as described [39].

The significantly different features (taxa) between two experimental groups were identified using three different algorithms, ANCOM [41] (v2.0), ALDEx2 [42] (v1.4.0), and LEfSe algorithm [43] with a default cutoff (the absolute log_10_ LDA score > 2.0 and *p <* 0.05 based on the Kruskal-Wallis test by ranks). ANCOM and ALDEx2 were designed to address compositionality issues inherent in the marker gene count data while the former has been shown to have a very low false discovery rate and comparable power to other methods [44,45]. The significant features reported in this study were based on the consensus results, with concordance from at least two of the three algorithms used. The R vegan package was used for microbial diversity analysis.

Microbial signatures or balances were identified using selbal (R version 3.6.1) with default parameters [45]. The randomForest (RF) R package (v4.6-14) was used. Both RF classification and regression models were performed in the study. The OTU table generated from QIIME1 was first collapsed to a genus level count table. The abundance data were then normalized based on total sum scaling. The RF parameters used were as follows: the number of trees in the forest (ntree) was set to 501 and the number of features randomly sampled at each node in a tree (mtry) was 13. The Z-score, or scaled mean decrease accuracy, was calculated and used to rank variable importance.

Microbial co-occurrence networks were constructed using FastSpar, an efficient and parallelizable implementation of the SparCC algorithm [46] for compositional data [47]. The OTU table was first collapsed to the genus level. Pseudo *p* values were computed using 100 randomized sets. The cutoff values for significant microbial interactions used were SparCC correlations absolute |R| ≥ 0.5 and *p* value ≤ 0.05. The network was then visualized using Cytoscape v3.6.1.

Metabolomic analysis: Untargeted metabolomics analysis of gut contents was conducted as described [48]. Briefly, individual samples were accurately weighed. The samples were then homogenized using a grinding mill and extracted. The samples were centrifuged at 12,000 rpm at 4 °C for 15 min; and 200 µL of the supernatant was transferred for LC-MS analysis. LC-MS was performed using a Ultimate 3000LC, Q Exactive (Thermo). Raw data were then extracted, peaks were identified and then quantified using the area under the curve method. Each compound was corrected in run/day blocks. The peak intensity data were then normalized and log transformed. Normalized data were analyzed using a Wilcoxon rank sum test to identify metabolites that may differ significantly among experimental groups. In addition, raw spectral data were analyzed using the XCMS pipeline with default parameters [36].

## 3. Results

### 3.1. Timing of Genistein Supplementation Determined Its Effect on Mammary Tumor Recurrence in the HFD Offspring

**Figure 1:**
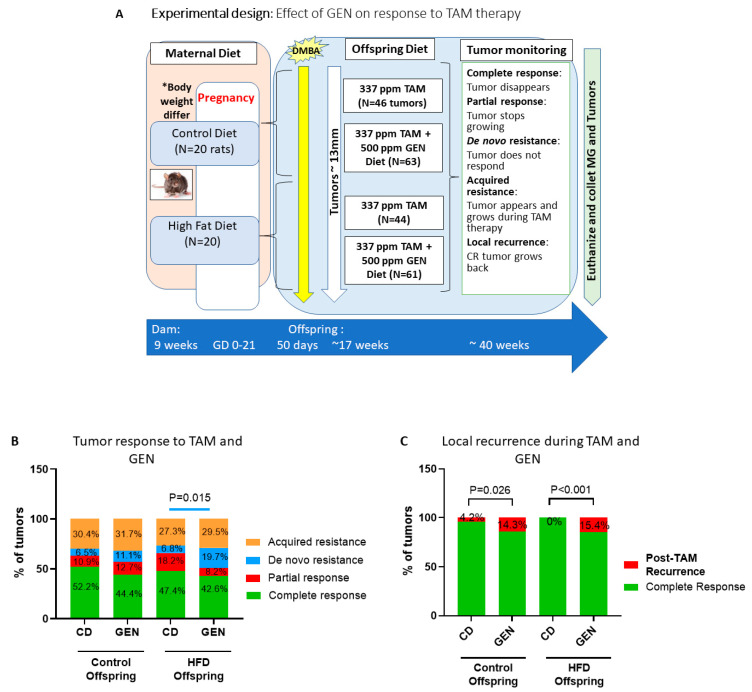
Genistein impairs response to tamoxifen therapy in the high fat diet (HFD) offspring. (**A**) Experimental design to study the effects of starting genistein feeding on tamoxifen responsiveness during therapy on local mammary tumor recurrence in the offspring of control or high fat diet (HFD) fed dams. (**B**) Mammary tumors were defined as complete response (green) when a tumor disappeared for at least six weeks, partial response (red) when a tumor stopped growing or shrunk, de novo resistance (blue) when a tumor never responded, and acquired resistance (orange) when tumor appeared and grew during tamoxifen treatment. Genistein supplementation during tamoxifen therapy significantly increased percentage of de novo resistance tumors among HFD offspring (*p* = 0.015). GEN (genistein supplemented) and CD (control diet without supplementation) Control/CD: *n* = 46 tumors; Control/GEN: *n* = 63 tumors; HFD/CD: *n* = 44 tumors, HFD/GEN: *n* = 61 tumors. (**C**) Genistein significantly increased tumor recurrence in the control offspring (control/GEN; out of 28 tumors, *p* = 0.026) and HFD offspring (HFD/GEN; out of 26 tumors); *p* < 0.001) compared to untreated groups (control/CD; out of 24 tumors, and HFD/CD; out of 20 tumors). Statistical significance according to Chi-Square test; * Dams fed HFD had significantly higher weight than control dams before mating (*p* = 0.009).

Mammary tumor response to tamoxifen therapy: The study design is shown in Figure 1A. In this study, genistein supplementation started at the same time with tamoxifen therapy. As we have previously reported [7], response of ER+ mammary tumors to tamoxifen therapy were similar in the offspring born to mothers fed a control diet and in the HFD offspring. In the control offspring, 32 tumors were present at the beginning of the treatment period, and 14 new, acquired tamoxifen-resistant tumors (30.4%) developed during the tumor monitoring period (mean ± SEM, length: 17.1 ± 1.7 weeks). Over half of all the tumors (52.2%) exhibited a complete response to tamoxifen (Figure 1B), and only 4.2% of these tumors recurred during tamoxifen treatment (Figure 1C). In the HFD group, 32 tumors were present at the beginning of the treatment period, and 12 new, acquired tamoxifen-resistant tumors (27.3%) developed during the tumor monitoring period (mean ± SEM length: 17.5 ± 2.1 weeks). Of the 44 tumors, 47.4% exhibited a complete response to tamoxifen (Figure 1B). None of these responding tumors recurred during tamoxifen treatment in the HFD offspring (Figure 1C).

Effects of genistein on mammary tumor response to tamoxifen therapy: In the control offspring, supplementation with genistein did not impact how tamoxifen affected ER+ mammary tumors, compared with non-supplemented control offspring (Figure 1B). Acquired tamoxifen-resistant tumor rate in the genistein treated control group (20 of 63 tumors; 31.7%) and the length of tumor monitoring period (16.1 ± 1.2 weeks) were similar to the non-genistein supplemented control offspring. However, the rate of recurrences among the responding tumors was three-fold higher (14.3%, *p =* 0.026, Chi-square test) during genistein treatment than without the treatment (Figure 1C).

In contrast to the control offspring, genistein significantly impaired responsiveness to tamoxifen in the HFD offspring (*p =* 0.015). The difference reflected an increase in the proportion of de novo tamoxifen-resistant tumors which was 6.8% in the non-supplemented HFD offspring, but almost three times higher in the genistein fed animals (19.7%) (Figure 1B). Complete (42.6% vs. 47.4%) and partial responses (8.2% vs. 18.2%) were lower in the HFD offspring supplemented with genistein than in the non-supplemented HFD offspring. Acquired resistant tumor rate in the genistein group was similar to that of the non-supplemented HFD offspring (29.5%; 18 of 61 tumors; length of tumor monitoring period: 16.5 ± 3.6 weeks). However, local mammary tumor recurrence during tamoxifen treatment increased from 0% to 15.4% by genistein in the HFD offspring (*p* < 0.001, Figure 1C).

### 3.2. Genistein after Tamoxifen Treatment Prevented Local Mammary Cancer Recurrence in the HFD Offspring

**Figure 2:**
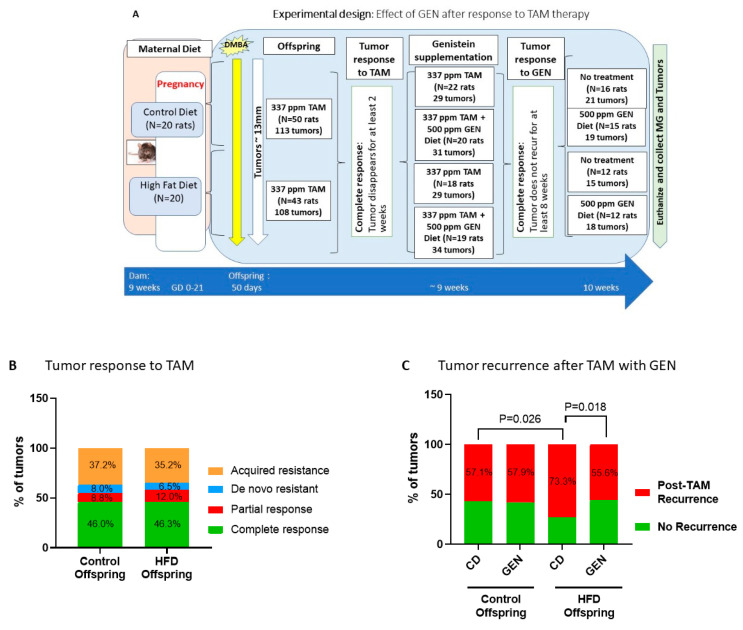
Genistein (GEN) after tamoxifen response prevents local recurrence in the HFD offspring. (**A**) Experimental design to study the effects of starting genistein feeding after complete response to tamoxifen on local mammary tumor recurrence in the offspring of control or high fat diet (HFD) fed dams. (**B**) Maternal exposure to HFD did not change initial response to tamoxifen (HFD, *n* = 108 tumors), compared to control offspring (*n* = 113 tumors). (**C**) After tamoxifen therapy, there was no difference in tumor recurrence among control offspring treated with GEN (con/GEN; out of 19 tumors) or not treated with GEN (control/CD; out of 21 tumors). Maternal HFD significantly increased (*p* = 0.026) mammary tumor recurrence after tamoxifen therapy (HFD/CD; out of 15 tumors), compared with control offspring. GEN supplementation significantly decreased (*p* = 0.018) tumor recurrence in the HFD offspring (HFD/GEN; out of 18 tumors). The data are shown as percentage of tumors. Statistical significance according to Chi-Square test.

The study design is shown in Figure 2A. In this study, genistein supplementation started after an offspring had exhibited a complete response to tamoxifen. Similar to Experiment 1, maternal HFD did not alter responses to tamoxifen (Figure 2B). However, after tamoxifen therapy ended, the risk of recurrence was 73.3% in the HFD offspring, and this was significantly higher than in the control offspring (57.1%; *p* = 0.026) (Figure 2C). Genistein significantly reduced the risk of local recurrences in the HFD offspring, with 55.6% of the genistein-treated HFD offspring exhibiting a local recurrence (*p* = 0.018). On the other hand, genistein supplementation had no impact on recurrence rates in the control offspring, i.e., 57.9% of the controls that received genistein after TAM therapy developed local recurrence.

### 3.3. Genistein Supplementation after Tamoxifen Therapy Altered the Gut Microbiota in the Control and HFD Offspring

**Figure 3:**
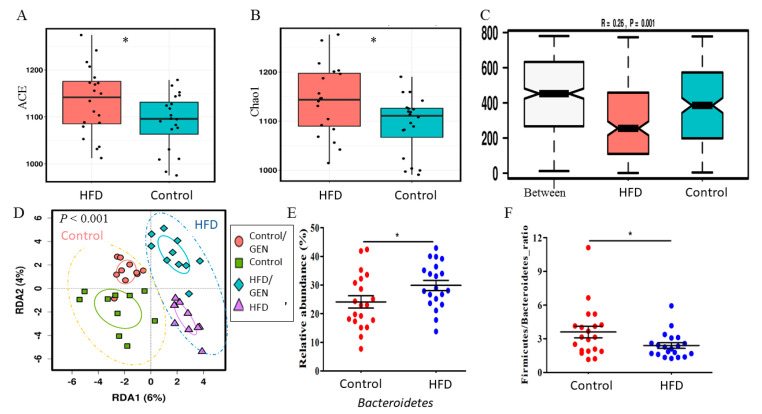
The maternal dietary status has a profound impact on the offspring microbiome. (**A**) The offspring of rat dams fed a maternal high-fat diet (HFD) had a significantly higher microbial richness, including Abundance-based Coverage Estimator (ACE), and (**B**) Chao1, compared with the offspring of dams fed a maternal control diet. The maternal dietary status also had a significant effect on beta diversity in the offspring, such as analysis of similarities (ANOSIM) based on Bray Curtis Dissimilarity (**C**) and redundancy analysis (RDA) based on weighted UniFrac metrics (**D**). (**E**) The abundance of the phylum *Bacteroidetes* was significantly increased in the HFD offspring as detected by ANCOM. (**F**) The *Firmicutes* to *Bacteroidetes* ratio was significantly reduced in HFD offspring. * *p <* 0.05.

Effect of maternal HFD on the offspring’s gut microbiota: Maternal HFD affected the gut microbial composition of the 8-month-old offspring which had all undergone several weeks of tamoxifen therapy, but at the time the fecal samples were collected, had been off from tamoxifen for at least five weeks. The offspring of HFD dams had a significantly higher level in two richness indices, i.e., Abundance-based Coverage Estimator (ACE) (Figure 3A) and Chao1 (Figure 3B; *p* < 0.05), compared with the offspring of dams fed a control diet. PERMANOVA results indicated that maternal HFD had a significant impact on offspring’s beta diversity based on either Bray Curtis dissimilarity (Pseudo F = 3.18; permutation-based *p =* 0.0001) or a weighted UniFrac distance metric (Adonis *p =* 0.0163).

**Figure 4:**
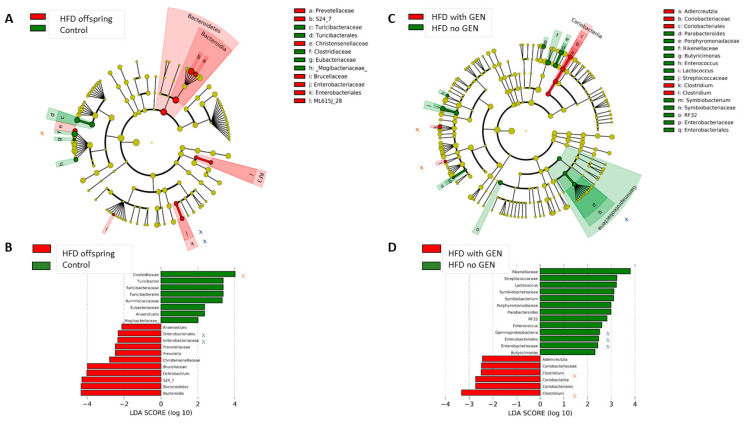
Differences in the gut microbiota in the offspring of control and high-fat diet (HFD) fed dams. Circular cladograms (**A**,**C**) show results from linear discriminant analysis effect size (LEfSe), presenting the identified OTUs distributed according to phylogenetic characteristics around the circle. Differentially expressed taxa are highlighted by colored and shaded circles. Each circle’s diameter is relative to abundance of taxa in the community. Results between control versus HFD offspring shown in (**A**), and between HFD offspring supplemented with genistein (GEN) or not supplemented shown in (**C**). (**B**,**D**) LEfSe results showing which bacteria were significantly different in abundance between the control and HFD offspring (**B**), or between HFD offspring supplemented with GEN after tamoxifen versus not supplemented (**D**). Bacteria marked as x or x are those that were significantly different between control and HFD offspring, and reversed by GEN in HFD offspring.

Additional multivariate analyses showed that maternal HFD impacted offspring’s gut microbial composition, compared with the control offspring (ANOSIM, Figure 3C; RDA, *p* = 0.0001, Figure 3D). When assessed using the analysis of composition of microbiomes algorithm (ANCOM) [42], the abundance of the family *Prevotellaceae* as well as the genus *Prevotella* was ~3-fold higher in HFD offspring than in control offspring. *Prevotella* is suggested to be a pathobiont that can participate in various human diseases by promoting chronic inflammation [49]. Other significant differences in the gut microbiota between the control and HFD offspring are shown in Figure 4A. Among the significantly increased inflammatory bacteria in the HFD offspring was the order *Enterobacteriales* (Wilcoxon *p =* 0.0025)*,* and family *Enterobacteriaceae* (*p =* 0.0041). These belong to the class *Gammaproteobacteria.* Maternal HFD also suppressed the abundance of SCFA-producing family *Clostridiaceae* (*p =* 0.0413). *Prevotella* belongs to *Bacteroidetes*, whilst *Gammaproteobacteria* and *Clostridiaceae are Firmicutes*. Consequently. HFD offspring exhibited a significantly higher abundance of *Bacteroidetes* (29.9% ± 7.9%) than the control offspring (24.1% ± 9.6%) (*p =* 0.038, LDA = 4.80; Figure 3E), resulting in a reduced *Firmicutes* to *Bacteroidetes* ratio (Figure 3F). Together, our data supports the notion that maternal HFD has a profound and sustained impact on the offspring’s gut microbial composition.

Effect of genistein supplementation on gut microbial signatures: Adult genistein supplementation altered the gut microbial composition both in the control and HFD offspring. In the HFD offspring only, genistein supplementation significantly reduced the abundance of the class *Gammaproteobacteria* (*p =* 0.0019) (Figure 4C). In this class, *Enterobacteriaceae* was significantly reduced (*p =* 0.0041). The abundance of *Clostridium*, a genus in *Clostridiaceae* family, was significantly increased (*p =* 0.034) by genistein supplementation in the HFD offspring (Figure 4D). Since maternal HFD led to an increased abundance of *Enterobacteriaceae* and inhibited *Clostridiaceae* in the offspring, compared with the control offspring (Figure 4A,B), genistein supplementation was able to reverse these changes. Other genistein supplementation induced changes in the HFD offspring are shown in Figure 4C,D.

**Figure 5:**
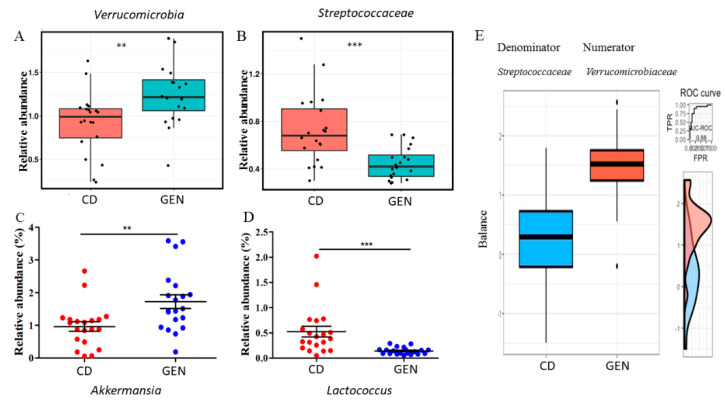
Genistein (GEN) supplementation affected the gut microbiome composition. (**A**) The phylum *Verrucomicrobia*, (**B**) the family *Streptococcaceae*, (**C**) *Akkermansia*, and (**D**) *Lactococcus* were significantly affected by adult GEN intake in the offspring regardless of their maternal dietary status. (**E**) A microbial signature selected by selbal with a strong predictive accuracy to distinguish the GEN supplementation status. The signature consisted of *Verrucomicrobiaceae* (Numerator) and *Streptococcaceae* (Denominator). The box plots represent the distribution of the balance values for each category. The right (vertical) panel of the figure represents the Receiver Operating Characteristics (ROC) curves with the Area under the ROC curve (AUC) values (top) and density curves (bottom) for each category. GEN: with the genistein supplementation; CD: without the supplementation. ** *p* < 0.01; *** *p* < 0.001.

In both the control and HFD offspring, genistein reduced beta diversity (permutation *p =* 0.0087) (Figure S1). The abundance of *Verrucomicrobia* (Figure 5A) was significantly higher in the control and HFD offspring fed genistein; this finding was confirmed by an independent analysis of feature tables generated by QIIME2 using a linear discriminant analysis (LDA) effect size (LEfSE) [43]. Approximately a 3-fold decrease in the abundance of *Streptococcaceae* was also detected by ANCOM by genistein supplementation (Figure 5B). The genus *Akkermansia* is the sole representative of *Verrucomicrobia*, and its abundance was significantly increased in rats fed genistein (Figure 5C). On the other hand, the abundance of *Lactococcus* was 0.27-fold lower in rats fed genistein than those fed control diet (Figure 5D). A log abundance ratio between *Verrucomicrobiaceae* and *Streptococcaceae* was significantly higher in the genistein supplemented than non-supplemented group (Area Under the Curve, AUC and cross validation AUC = 0.88 and 0.71, respectively) (Figure 5E). 

**Figure 6:**
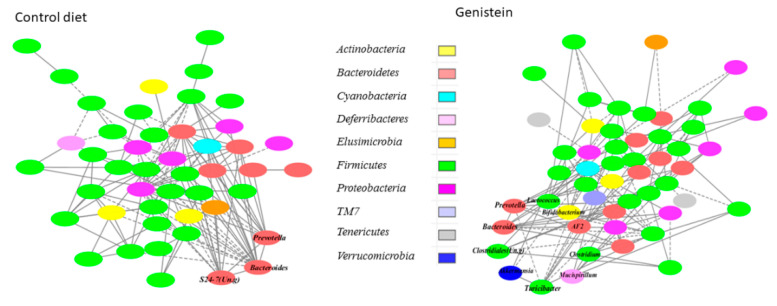
Effect of genistein (GEN) supplementation on microbial interaction networks inferred using the SparCC algorithm. Solid line: positive correlation; dashed line: negative correlation. The color of each node (genus) represents the phylum to which this genus is assigned.

Effect of genistein supplementation on microbial interaction networks: Microbial interaction networks were inferred using a Sparse Correlations for Compositional data (SparCC)-based algorithm [46]. While the number of input genera between the networks inferred was similar in the genistein supplemented and non-supplemented groups, the network in genistein-supplemented rats had fewer interactions compared with the non-supplemented rats (Figure 6). The basic microbial interactions, approximately 28% of the total, were preserved in the networks of both the genistein-supplemented and non-supplemented animals. For example, the basic structure of the three modules with *Bacteroides*, *Bifidobacterium* and *Prevotella* as hubs existed in both networks with strong positive correlations (R ≥ 0.75). *Bifidobacterium* also was strongly and positively correlated with *Allobaculum* in the networks of genistein-supplemented and non-supplemented rats (R = 0.86 and 0.95, respectively; *p <* 0.001). However, genistein supplementation altered some of the microbial interactions. The module with an unclassified genus in S24-7 as a hub was disrupted by the genistein supplementation. In contrast, a module centered on an unclassified genus in the order *Clostridiales* was detected only in the genistein supplemented network. While the interaction between *Lactococcus* and *Streptococcus* was maintained in the genistein-supplemented animals, genistein promoted the interactions between *Lactococcus* and *Anaerostipes* and two unclassified genera in *Peptococcaceae* and *Erysipelotrichaceae*. Genistein supplementation enhanced network interactions between the genus *AF12* in the family *Rikenellaceae* and several genera in *Lachnospiraceae* (R > 0.60; *p <* 0.01), and between *Allobaculum* and *Akkermansia* (R = 0.81; *p <* 0.01). In the HFD offspring, genistein promoted the interaction of *Akkermansia* with other genera, such as *Bifidobacterium* and *Turicibacter*.

### 3.4. Rats Exhibiting Local Mammary Tumor Recurrence Had a Different Gut Microbiota Composition than Rats Remaining Recurrence Free

**Figure 7:**
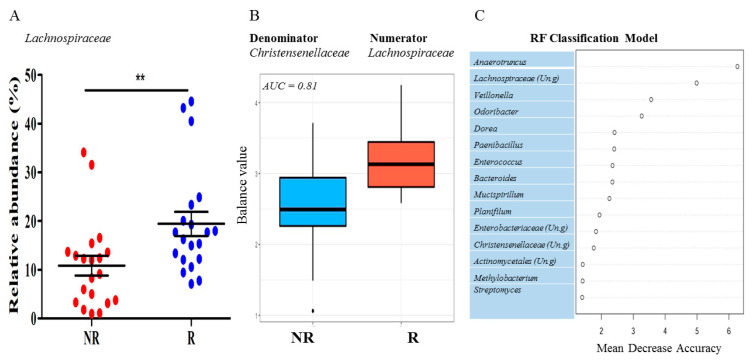
Microbial signatures associated with local recurrence of the estrogen receptor positive breast tumor after tamoxifen treatment in a rat model. (**A**) The abundance of the family *Lachnospiraceae* was significantly higher in rats with tumor recurrence. (**B**) A microbial signature consisting of *Lachnospiraceae* (numerator) and *Christensenellaceae* (denominator) accurately predicted the recurrence status. AUC = Area under the ROC (receiver operating characteristics) curve. R: with local recurrence; NR: without local recurrence. (**C**): The top 20 microbial predictors that contributed to the classification accuracy between the groups with (R) or without (NR) local recurrence. (Un.g) = unclassified genus. ** *p <* 0.01.

We explored possible differences in the gut microbiota between rats that exhibited local recurrence and those that remained recurrence free, regardless of maternal dietary exposure or genistein supplementation. The family *Lachnospiraceae* was the only taxon that showed a significant difference in the relative abundance for the local recurrence status. The rats with local tumor recurrence had a significantly higher level of *Lachnospiraceae* than those without tumor recurrence (Figure 7A). The log ratio between two families in the class of *Clostridia*, i.e., *Lachnospiraceae* (Numerators) and *Christensenellaceae* (Denominator), had a significant predictive accuracy for the local recurrence (AUC = 0.81), detected by the selbal algorithm [47]. As shown in Figure 7B, the abundance of *Lachnospiraceae* was higher than that of the *Christensenellaceae* in the gut microbiota of animals with local mammary tumor recurrence. *Anaerotruncus* and an unclassified genus in *Lachnospiraceae* were among the most important features in contributing to the classification accuracy between the local recurrence status, as detected by the RF algorithm (Figure 7C).

### 3.5. Genistein Supplementation Altered Microbial Metabolism

Untargeted metabolomic data were first annotated against the fecal fraction of the Human Metabolome Database (HMDB). Maternal diet had a limited effect on the offspring’s gut metabolome. No metabolite displayed a significant difference between the offspring born to control or HFD fed dams at a false discovery rate or FDR < 0.1. Nevertheless, 51 metabolites were significant at a nominal *p <* 0.05 (Table S2). Of these, genistein reversed the increased levels of N-acetylvaline (HFD vs. control offspring: fold-difference = 1.70, *p =* 0.038) in the HFD offspring (genistein-supplemented vs. non-supplemented: fold-difference = 0.39, *p =* 0012). High N-acetylvaline levels have been linked to increased all-cause and cardiovascular disease mortality [50]. Significantly enriched pathways between the control and HFD offspring were assessed by the Lilikoi pipeline [51]. Of the cancer hallmark pathways, the Warburg effect linked to cancer cell metabolism was significantly enriched in the HFD compared with the control offspring (the gain ratio or GR = 0.42; Table S3).

**Figure 8:**
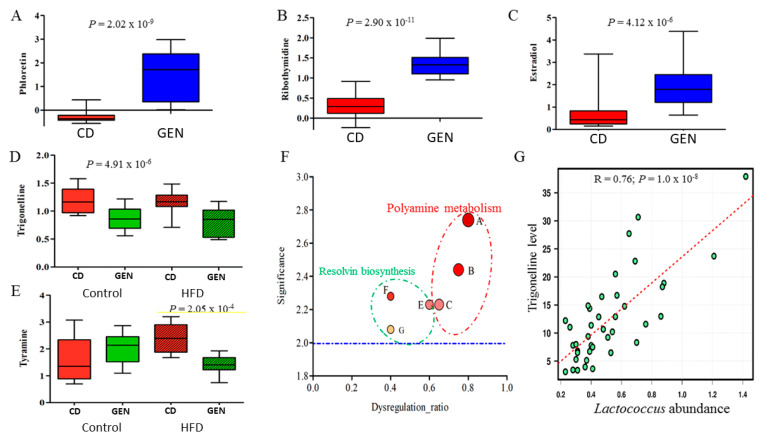
The gut metabolome is significantly impacted by genistein (GEN) supplementation. The level of select fecal metabolites that were significantly increased by GEN supplementation, compared with non-supplemented state, regardless of maternal dietary exposure: (**A**) phloretin, (**B**) ribothymidine, and (**C**) estradiol. Abundance of some metabolites were similarly reduced by GEN supplementation in the control and HFD offspring: (**D**) trigonelline; and some were reduced only in the HFD offspring (**E**): tyramine. (**F**) Additional select pathways which were significantly affected by GEN supplementation only in the HFD offspring. The selected pathways were: A, spermine biosynthesis; B, spermidine biosynthesis; C, spermine and spermidine degradation; E, aspirin triggered resolvin E biosynthesis, the yellow line is designed to highlight the significance; F, aspirin triggered resolvin D biosynthesis; and G, resolvin D biosynthesis. Significance was defined as -log_10_ *p* value. The blue dashed line represented significance of *p =* 0.01. The Dysregulation ratio was defined as the number of dysregulated to the number of total metabolites detected in the dataset that were assigned to a given pathway. (**G**) The linear correlation between the relative abundance of the genus *Lactococcus* and the fecal trigonelline level.

When genistein-induced changes in metabolites were assessed regardless of maternal diets, a total of 18 metabolites differed significantly in relative peak intensities between genistein-supplemented and non-supplemented rats (FDR < 0.1; Table S4). A significantly higher level of naringenin, phenylalanylproline, phloretin (Figure 8A), ribothymidine (Figure 8B), and tyrosol were detected in genistein-supplemented rats. Remarkably, the genistein supplementation resulted in a 240-fold increase in the level of phloretin (FDR = 5.47 × 10^−7^). Moreover, the level of estradiol was increased by almost 3-fold due to the genistein supplementation (FDR = 6.65 × 10^−4^, Figure 8C). On the other hand, genistein supplementation significantly decreased the level of trigonelline (FDR < 0.1; Figure 8D) as well as bilirubin, stercobilinogen, and thiosulfate (Table S4). These metabolites were among the most important predictors identified by the RF classification model that accurately distinguished animals supplemented with genistein from non-supplemented animals.

Raw spectral data of untargeted metabolomics was independently analyzed using the XCMS pipeline against the METLIN database [52]. A total of 120 metabolites displayed a significant difference in normalized peak intensities between genistein supplemented and non-supplemented groups (FDR < 0.10). For example, the level of S-methyl-5’-thioadenosine was approximately 4.5-fold higher in the rats fed genistein than those without the supplementation (FDR = 2.07 × 10^−4^), whilst the level of α-tocopherol was significantly lower in genistein fed rats (FDR = 1.44 × 10^−3^). Overall, there were at least eight pathways that were significantly altered between the genistein supplemented and non-supplemented groups. Among these pathways, morphine and glucocorticoid biosynthesis were most impacted by the genistein supplementation. Spermine and spermidine biosynthesis and α-tocopherol degradation were also among the significantly altered pathways (*p =* 2.1 × 10^−4^). Genistein enriched metabolic pathways identified by the Lilikoi pipeline included arginine and proline metabolism (Table S3).

Metabolic pathways that were only altered in the HFD offspring by genistein supplementation. Some of the gut metabolic pathways were affected by genistein only in the HFD offspring: they included tyramine, polyamine metabolism and resolvin biosynthesis. The level of tyramine, a compound in the disulfiram action pathway, was significantly decreased by genistein in the offspring of HFD fed dams (FDR = 0.0258; Figure 8E), but not in the control offspring. Moreover, at least three pathways related the pro-resolving phase of inflammation, including resolvin D biosynthesis and aspirin triggered biosynthesis of resolvin D and E1, and polyamine metabolism were increased by genistein supplementation in the offspring of HFD fed dams (Figure 8F,G).

Other pathways affected by genistein supplementation only in the HFD offspring included the disulfiram action and nicotinate and nicotinamide metabolism (GR = 0.57). Moreover, amino sugar metabolism, carnitine synthesis, glycine and serine metabolism, and ibuprofen action pathway were also significantly enriched (GR > 0.46; Table S3).

The microbiome taxon–metabolite associations: Finally, the microbiome taxon–metabolite associations were evaluated using Pearson correlation analysis. There were at least 10 genera that showed a significant correlation with the level of sterocobilinogen (*p <* 0.05), which was significantly altered by the genistein supplementation. The top three genera based on the highest correlation coefficient (R) were *Anaeroplasma* (R = 0.62; *p =* 2.1 × 10^−5^), *Turicibacter* (R = 0.46; *p =* 3.2 × 10^−3^), and *Lactococcus* (R = 0.45; *p =* 3.2 × 10^−3^). Similarly, among the nine genera showing a significant association with the level of trigonelline, the genus *Lactococcus* was the strongest (R = 0.76; *p =* 1.0 × 10^−8^; Figure 8G), followed by an unclassified genus in *Leuconostocaceae* (R = 0.44; *p =* 4.7 × 10^−3^). A RF regression model analysis suggests that there exists a moderate but significant correlation between this metabolite and microbiome variables, with the average regression error of 6.6%. *Lactococcus*, *Akkermansia*, and an unclassified genus in *Erysipelotrichaceae* were among the top three important microbiome features contributing to the regression accuracy.

### 3.6. Genistein Activated Anti-Tumor Immune Responses in Tamoxifen Responding, But Not Resistant Tumors

Effect of genistein on inflammatory and immune markers in the tamoxifen responding mammary tumors: The effects of maternal HFD on offspring’s immune genes were evaluated in tamoxifen-responding and resistant tumors. In the tamoxifen sensitive tumors, we found that maternal intake of HFD upregulated markers of immunosuppression, i.e., *Foxp3* (*p =* 0.013) and *Pd1* (*p =* 0.05) significantly, and *Tgfβ1* (*p =* 0.07) and *Il-6* (*p =* 0.08) marginally. HFD offspring also exhibited a non-significantly suppressed *Cd8a* (*p =* 0.07), compared with control offspring (Figure S2). Genistein supplementation significantly upregulated *Cd8a* (*p <* 0.001) (Figure S2A), and suppressed *Foxp3* (*p =* 0.006) (Figure S2B), *Tgfβ1*(*p* < 0.001) (Figure S2C), *Pd1* (*p =* 0.021) (Figure S2D), *Ctla4* (*p =* 0.024) (Figure S2F) and *Il6* (*p =* 0.014) (Figure S2G) expression in the HFD offspring. In the control offspring genistein did not affect any of the immune genes in the responding tumors of the control offspring.

In the tamoxifen-resistant tumors (Figure S2), the only change genistein induced in the HFD offspring was an increase in *Il6* expression (*p =* 0.037): this change is the opposite to the reduction in *Il6* by genistein in sensitive tumors. Tamoxifen-resistant mammary tumors in the control rats did not respond to genistein. Several of the immune genes were expressed at a significantly lower level in the resistant than sensitive mammary tumors in the HFD offspring not treated with genistein: *Foxp3* (*p* = 0.012), *Tgfb1* (*p <* 0.001), *Ctla4* (*p* = 0.023) and *Il6* (*p* = 0.018). In the control offspring, resistant tumors expressed significantly lower level of *Cd8* (*p* = 0.011; two-way ANOVA), *Tgfb1* (*p <* 0.036; two-way ANOVA), and *Pdl1* (*p =* 0.003, two-way ANOVA).

Effect of genistein on inflammatory and immune markers in the recurring mammary tumors: Finally, in the recurring tumors, none of immune genes were differentially expressed between the control and HFD offspring. Further, genistein had no effects on these genes in the HFD offspring. In the control offspring, genistein significantly upregulated *Tgfβ1* (*p =* 0.007) and tended to increase Pdl1 (*p =* 0.06) in the recurring tumors (Figure S3).

## 4. Discussion

The findings in humans indicate that soy food intake reduces the risk of breast cancer recurrence [53,54]. This likely reflects the accumulative effect of lifetime soy consumption, since in many preclinical studies only animals that started consuming genistein before mammary tumors were present exhibited reduced tumorigenesis [29,55] or reduced local mammary tumor recurrence [26]. Starting genistein supplementation with tamoxifen therapy increased the risk of local recurrences in an animal model [26]. Here we confirmed the earlier findings and showed that starting genistein intake with tamoxifen therapy impaired responsiveness to this antiestrogen therapy in the HFD offspring and increased the risk of local recurrence both in the control and HFD offspring. However, starting genistein supplementation after response to tamoxifen and continuing genistein intake after tamoxifen ended prevented an increased risk of local recurrence in the HFD offspring. If translatable to humans, these findings suggest that if a patient has not consumed soy foods before breast cancer is diagnosed, she/he should not start consuming it at the beginning of tamoxifen therapy. However, when the tumor responds to tamoxifen, starting genistein intake may prevent recurrences.

Genistein binds and activates the three different types of estrogen receptors (ERα, ERβ and GPER) [56,57], possibly explaining the multiple potential mechanisms of action proposed for genistein to alter breast cancer risk and risk of recurrence. For example, immune cells express all three estrogen receptors [58,59]. It has been shown that genistein inhibits NFkB, which regulates IL6 and other inflammatory cytokines [60]. We found here that genistein inhibited *Il6* expression in the mammary tumors in the HFD offspring. GEN also upregulates IFNγ [61] and IL2 [62], both of which stimulate CD8+ T effector cell activation. In addition, GEN suppresses *Tgfβ1*, likely inhibiting immunosuppressive FOXP3 expressing T regulatory (Treg) cells. In our earlier study, lifetime genistein intake upregulated *Cd8a* expression in the TME and inhibited *Foxp3* and *Tgfβ1* [26]. Similar results were obtained here in the HFD offspring. Thus, in utero HFD exposure may sensitize the mammary tumor for genistein’s anti-inflammatory and anti-tumor immune responses which are seen in non-HFD exposed animals only if genistein intake begins before tumors are present [26].

Genistein might also affect the immune cells in the TME in the HFD offspring through its effect on the gut microbiota, the main immune organ in the mammalian body. The gut expresses the three estrogen receptors, especially ERβ [63,64]. Several earlier studies have shown that intake of genistein in animals [29,65] and soy foods in humans [66] alters the gut microbiota and fecal metabolites, although the alterations are different across these studies. In a study done using immunocompromised Rag2-/- mice which were humanized with fecal microbiota transplants from breast cancer patients, a genistein-supplemented diet increased the phylum *Verrucomicrobia* and the species *Akkermansia muciniphila* [29]. Similar changes were observed in our study here in the rats fed genistein, regardless of maternal dietary exposure. However, another prominent change induced by genistein in Rag2-/- mice was an increase in the genus *Lactococcus* [29]: *Lactococcus* was significantly reduced in our study in genistein-fed rats. Since rats in our study were exposed earlier to tamoxifen, some differences in our study and the study by Paul et al. [29] are expected. Endocrine therapy affects the gut microbiota [67]. It is possible that in our study the well-known health benefits of an increase in the abundance of *Akkermansia muciniphila*, which include improved glucose and lipid metabolism and intestinal immunity [68], and improved response to immune checkpoint inhibitors against different cancers [69], were masked by a reduction in *Lactococcus* which also has many health benefits, such as reduced risk of various cancers [70]. Together these changes may explain why genistein did not impact local mammary tumor recurrences in the control offspring.

To understand why genistein reduced local mammary tumor recurrence in the HFD offspring, we identified genistein-induced changes in the gut microbiota that were present only in the HFD offspring. As expected from prior studies, the composition of the gut microbiota between control and HFD offspring was different. The gut microbial richness was significantly higher in the HFD offspring. In earlier studies, maternal obesity has been linked to increased [17] or reduced microbiome diversity [71], or no changes in the diversity index among the offspring [21]. In the study Srinivasan et al. [71] that reported reduced microbiome diversity by maternal HFD, control dams were fed a diet high in soluble fiber and plant-chemicals, both of which tend to increase microbial diversity [72]. Since the obesity-inducing HFD was low in phytochemicals and contained insoluble cellulose, reduced microbial diversity in the HFD offspring in the Srinivasan et al. study [71] might reflect fetal exposure to phytochemicals and soluble fiber in the control diet. Although a rule of thumb is that cancer risk is inversely linked to high microbial diversity [73], there are exceptions to this rule. For example, the microbial diversity is reported to be significantly higher in postmenopausal breast cancer patients than in postmenopausal controls [74], or in cervical cancer patients than in healthy controls [75].

Other differences between the control and HFD offspring were an increase in the abundance of strongly inflammatory *Prevotella, and Enterobacteriaceae* and *Enterobacteriales* of the class *Gammaproteobacteria.* *Prevotella* increase the release of inflammatory mediators from immune and other cells and are therefore suggested to promote chronic inflammation and increase the risk of diseases in which inflammation plays an important role, including cancer [49]. The family *Enterobacteriaceae* possess lipopolysaccharide molecules which are known triggers of inflammatory responses [33]. HFD offspring also exhibited a significant reduction in anti-inflammatory, SCFA-producing *Clostridiaceae* [34]. In our study, control and HFD offspring consumed the same diet since being born and were postnatally exposed to the same treatments and environmental conditions. Thus, the differences in the composition of the gut microbiota in the HFD offspring reflect permanent effects of the early life dietary exposures on their gut microbiota. Importantly, the increased abundance of *Enterobacteriaceae* and reduction in *Clostridiaceae* were reversed by genistein supplementation in the HFD offspring, but genistein did not alter these microbes in the control offspring.

One of the central roles of the gut microbiota is to synthesize, modulate, and degrade various metabolites, making it a functional complement to host metabolism. These functions are particularly critical for dietary components that the host genes cannot synthetize or metabolize, such as SCFAs [76,77]. Further, more than 50% of all the metabolites in feces and urine are derived from or modified by the gut microbiota [78]. There were only few gut microbiota metabolites that were different between the HFD and control offspring. Of these, genistein reversed the increased levels of N-acetylvaline. Interestingly, high N-acetylvaline levels in the serum are linked to increased all-cause and cardiovascular disease mortality [50]. The main gut metabolic change in the pathway level in the HFD offspring involved the tumor Warburg effect: this pathway was significantly enriched in the HFD offspring compared with the controls. The Warburg effect provides energy to rapidly proliferating cancer cells and allows increased glucose uptake and fermentation of glucose to lactate. It is possible that the Warburg effect is linked to the increased risk of recurrence in the HFD offspring.

In the HFD offspring, but not in the control offspring, genistein supplementation impacted polyamine metabolism, especially spermine and spermidine (aspirin) biosynthesis and degradation. Observed changes in these and pro-resolving inflammatory pathways, such as resolvin biosynthesis, provide further evidence that supports the well-documented anti-inflammatory effect of genistein. Further, spermidine is linked to reduced carcinogenesis [79]. Soy foods, among many other foods, contain spermidine. Since spermine and spermidine levels in the gut in the genistein consuming rats were more elevated in the HFD than control offspring, indicating that although some of the effects of genistein on the gut microbiome were not dependent on the maternal diet, genistein also had unique effects seen only in the HFD offspring. Genistein also reduced tyramine, a biogenic amine produced via the enzymatic decarboxylation and deamination of amino acid tyrosine, in the HFD offspring but not in the controls. Since high tyramine levels are carcinogenic [80], the reduction in tyramine in the HFD offspring may be linked to their reduced risk of local mammary tumor recurrence by genistein.

Many of the gut microbiota metabolite changes induced by genistein were seen both in the control and HFD offspring, including a 240-fold increase in the phloretin level. Phloretin is a phenolic compound which, besides being generated by the gut microbiota, is present in many fruits, such as the apple. It has a wide variety of biological activities that can result a reduction in carcinogenesis, including acting as an antioxidant, being anti-inflammatory, blocking cyclins and cyclin-dependent kinases and inducing apoptosis by activating mitochondria-mediated cell death [81]. Other metabolites that were increased included ribothymidine and estradiol. Many earlier studies have investigated if genistein alters circulating estradiol levels. A meta-analysis of the data from 11 studies in premenopausal women and 35 studies in postmenopausal women did not find any significant change in circulating estradiol levels linked to a consumption of soy foods [82]. The increase in fecal estradiol levels in our study may result from an increased abundance of microbes that encode enzymes which promote estrogen release from its conjugated forms. Future studies should evaluate the activity of those enzymes, such as ß-glucuronidases, in the fecal samples. The genistein-induced reduction in gut trigonelline levels may have some practical implications. Trigonelline is a niacin-related compound, a natural constituent of coffee accounting for approximately 1% dry matter in roasted beans. It promotes the growth of MCF-7 mammary tumor cells [83] but inhibits the growth of pancreatic cancer cells [84]. Moreover, the RF regression model identified *Lactococcus* and *Akkermansia* as the two most important predictors for gut trigonelline levels. If trigonelline is pro-tumorigenic, it is possible to use prebiotics to regulate its level via the control of the gut *Lactococcus* expansion. A genistein associated increase in phloretin and a decrease in trigonelline, regardless of maternal diets, may contribute to the overall biological functions of this phytochemical, rather than explain the reduction in local mammary tumor recurrence in the HFD offspring.

To investigate whether the changes in the gut microbiota in the control and HFD offspring are linked to the risk of recurrence, we examined the gut microbiota in the rats that exhibited a local recurrence versus those remained recurrence free. The main finding was that the abundance of *Lachnospiraceae* was higher in the gut microbiota of animals with local mammary tumor recurrence, regardless of whether they were supplemented with genistein or not supplemented. Although certain members of *Lachnospiraceae* are among the main producers of SCFAs [85], some are associated with intestinal diseases, such as the irritable bowel syndrome (IBS) [86], metabolic disturbances [87], and liver diseases [88]. To further complicate the beneficial versus harmful role of *Lachnospiraceae,* its abundance in the gut microbiota was recently linked to fewer adverse effects caused by cancer radiation treatment [89]. Genistein supplementation enhanced network interactions between several genera in *Lachnospiraceae* and the genus *AF12* in the family *Rikenellaceae.* AF12 may exert beneficial effects of healthy diet and exercise on obese individuals [90], and therefore genistein might reverse the potential harmful effects of *Lachnospiraceae* on the risk of mammary cancer recurrence. Future studies will determine if the log abundance ratio between *Lachnospiraceae* and *Christensenellaceae* is translatable as a predictive biomarker for breast cancer recurrence in humans.

In conclusion, this study confirms our earlier finding that starting genistein intake with tamoxifen therapy blocks the ability of tamoxifen to inhibit mammary tumorigenesis [26]. This adverse outcome was also seen in the HFD offspring. However, if genistein intake started after a response to tamoxifen therapy was seen and continued when the therapy ends, genistein prevented the increase in local mammary tumor recurrence in the HFD offspring. In these offspring, the reduction in mammary tumor recurrence by genistein was proceeded by a reversal of gene expression indicative of tumor immunosuppression. Genistein significantly ameliorated maternal HFD-induced alterations in the offspring’s gut microbiota, including inhibiting the abundance of inflammatory *Enterobacteriaceae* and upregulating SCFA and anti-inflammatory *Clostridiaceae.* Genistein intake also modulated gut metabolites, particularly those related to polyamine metabolism and pre-resolving phase of inflammation in the HFD offspring, possibly contributing to its effect in reducing mammary tumor recurrence.

## Data Availability

All raw sequence data were deposited to NCBI SRA database with free public access (Accession# PRJNA660603). The data regarding microbiome feature and OTU tables and raw data for untargeted metabolome analysis presented in this study are openly available in Mendeley data at http://dx.doi.org/10.17632/pd8j2ttjyw.1 and http://dx.doi.org/10.17632/s4y6y7vz94.1, respectively.

## Supplementary Materials

The following are available online at https://www.mdpi.com/2072-6643/13/1/201/s1, Figure. S1: The genistein supplementation significantly reduced Chao 1 microbial richness. *n* = 20 per group. *p* < 0.05; Figure. S2: Genistein reverses markers of immunosuppression in the tamoxifen responsive mammary tumors in the HFD offspring. Gene expression of *Cd8a* (**A**), *Foxp3* (**B**), *Tgfβ1* (**C**), *Pd1* (**D**), *Pdl1* (**E**), *Ctla4* (**F**), *Il6* (**G**) in mammary tumors of control and HFD offspring treated with tamoxifen and supplemented with genistein. Maternal HFD significantly increased expression of *Foxp3* (*p* = 0.013), *Pd1* (*p* = 0.05) and non-significantly increased *Tgfb1* (*p* = 0.07), *Il6* (*p* = 0.08) and decreased *Cd8* (*p* = 0.07) in responding tumors. Maternal HFD did not significantly change expression of any of the genes in resistant tumors. Genistein did not change expression of any gene in responding and resistant tumors of control offspring compared to untreated control. Among HFD offspring, genistein supplementation increased expression of *Cd8* (*p* < 0.001) and decreased expression of *Foxp3* (*p* = 0.006), *Tgfβ1* (*p* < 0.001), *Pd1* (*p* = 0.021), *Ctla4* (*p* = 0.024) and *Il-6* (*p* = 0.014) in responding tumors. In resistant tumors, genistein increased expression of *Il-6* (*p* = 0.037) among HFD offspring. Differences according to t test when comparing the effects of Maternal exposure only and Two-Way ANOVA followed by Holm-Sidak test when comparing the effects of Maternal exposure and offspring treatment. Means ± SEM, *n* = 2–11 are shown; Figure. S3: Effects of genistein supplementation on recurring tumors. Gene expression of *Cd8a* (**A**), *Foxp3* (**B**), *Tgfβ1* (**C**), *Pd1* (**D**) and *Pdl1* (**E**) in recurring mammary tumors of control and HFD offspring treated with tamoxifen and supplemented with genistein after tamoxifen response. Genistein supplementation significantly and non-significantly increased expression of *Tgfβ1* (*p* = 0.007) and *Pdl1* (*p* = 0.06), respectively, in the control offspring compared to untreated control. Genistein did not change expression of *Cd8*, *Foxp3* and *Pd1* in both control and HFD offspring and *Tgfb1* and *Pdl1* in HFD offspring. Differences according to Two-Way ANOVA followed by Holm-Sidak test for *Foxp3* and *Tgfb1* and t test for *Pdl1*. Means ± SEM, *n* = 7–9 are shown; Table S1: Primers used in quantitative real-time PCR; Table S2. Metabolites significantly dysregulated in gut contents of the offspring of dams fed a high-fat diet (HFD). *n* = 20 per group. CD: offspring of the dams fed a control or low-fat diet; Table S3. The Lilikoi algorithm identified biological pathways significantly enriched in the fecal samples in the offspring of dams maternal high-fat diet or with or without genistein supplementation. HFD: The offspring of dams fed a maternal high-fat diet. CD: the offspring of dams fed a maternal control diet. CD/GEN: rats from control dams with the genistein supplementation. CD/CD: rats from control dams without the genistein supplementation. HFD/GEN: rats from HFD dams with the genistein supplementation. HFD/CD: rats from HFD dams without the genistein supplementation. GEN: with genistein supplementation. CD: without genistein supplementation; Table S4. Top 20 important metabolites based on mean decrease accuracy identified by the Random Forest algorithms contributing to the classification of the genistein supplementation status.
