# Supplementary material for: Genistein Reduces the Risk of Local Mammary Cancer Recurrence and Ameliorates Alterations in the Gut Microbiota in the Offspring of Obese Dams
Source: Nutrients. 2021 Jan 11;13(1):201. doi: 10.3390/nu13010201 (PMC7827465; doi:10.3390/nu13010201)
Supplement: Supplementary file 1 [file nutrients-13-00201-s001.pdf]

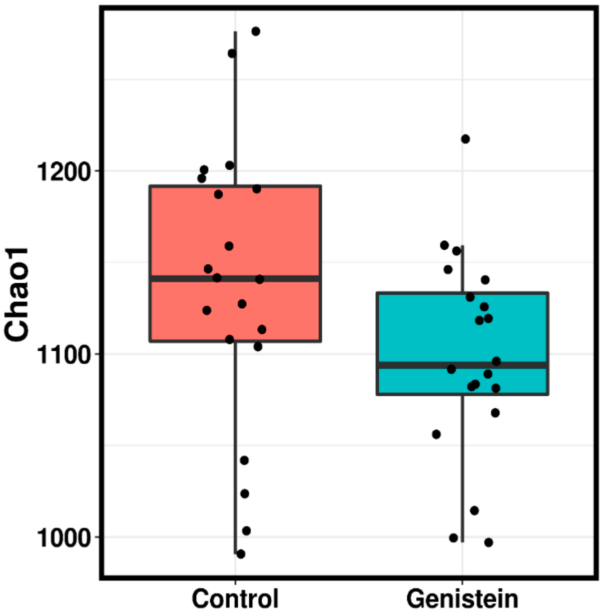

Supplementary Fig. S1. The genistein supplementation significantly reduced Chao 1 microbial richness. N = 20 per group. P < 0.05.

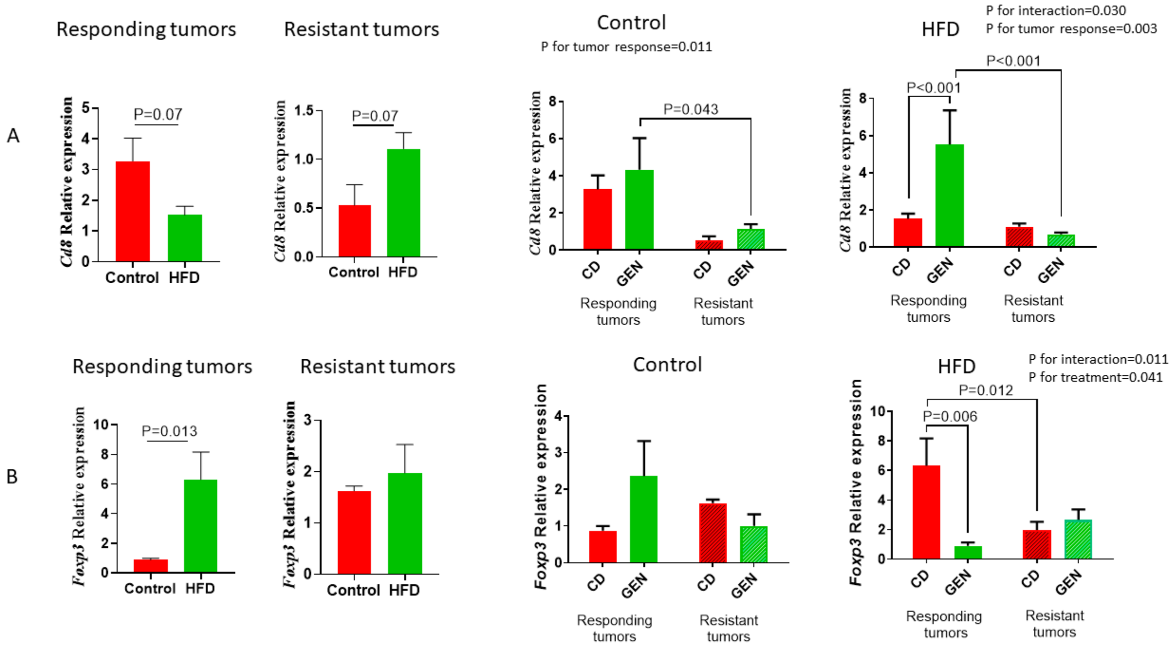

C

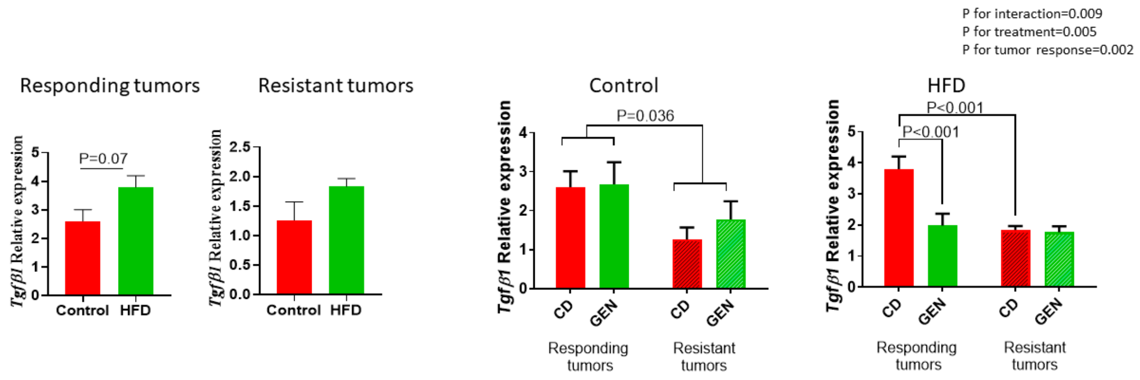
P for interaction=0.009
P for treatment=0.005
P for tumor response=0.002
Responding tumors
Resistant tumors
Control
HFD
P=0.07
Tgfβ1 Relative expression
Control HFD
P=0.036
P<0.001
P<0.001
CD
GEN
Responding tumors
Resistant tumors

D

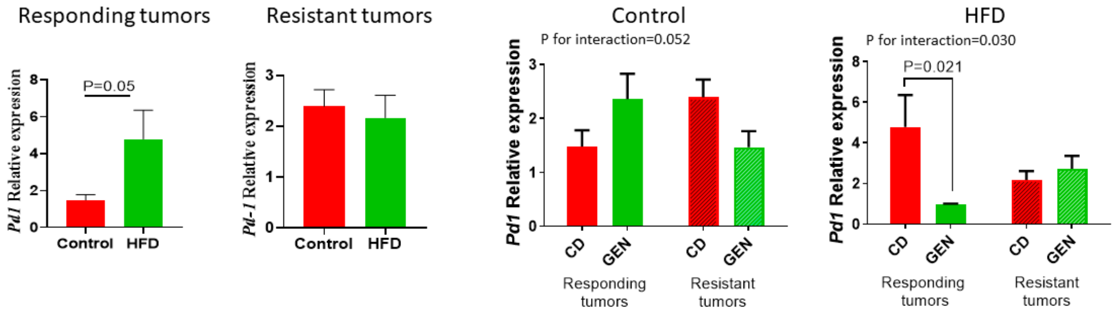
Responding tumors
Resistant tumors
Control
HFD
P for interaction=0.052
P for interaction=0.030
P=0.05
P=0.021
Pd1 Relative expression
Pd-1 Relative expression
Control HFD
CD
GEN
Responding tumors
Resistant tumors

F

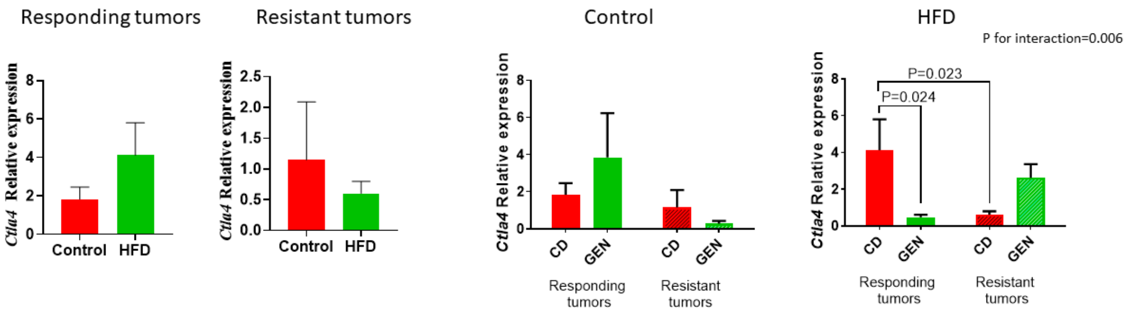

G

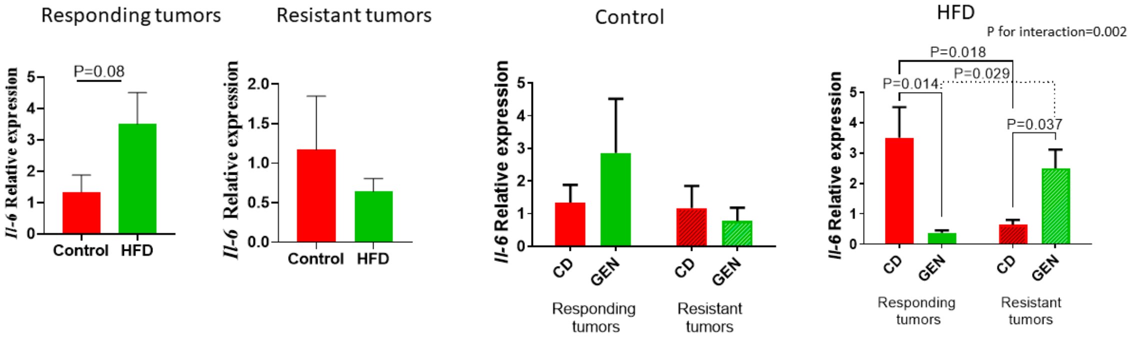

E

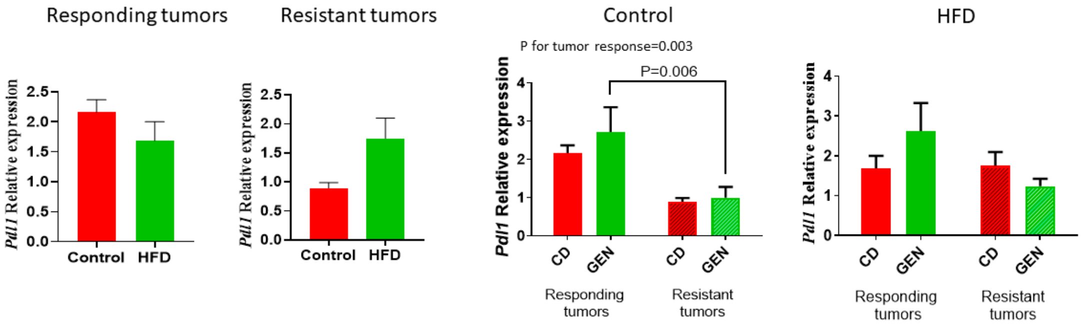

Supplementary Fig. S2. Genistein reverses markers of immunosuppression in the tamoxifen responsive mammary tumors in the HFD offspring. Gene expression of *Cd8a* (A), *Foxp3* (B), *Tgfβ1* (C), *Pd1* (D), *Pdl1* (E), *Ctla4* (F), *Il6* (G) in mammary tumors of control and HFD offspring treated with tamoxifen and supplemented with genistein. Maternal HFD significantly increased expression of *Foxp3* (P=0.013), *Pd1* (P=0.05) and non-significantly increased *Tgfb1* (P=0.07), *Il6* (P=0.08) and decreased *Cd8* (P=0.07) in responding tumors. Maternal HFD did not significantly change expression of any of the genes in resistant tumors. Genistein did not change expression of any gene in responding and resistant tumors of control offspring compared to untreated control. Among HFD offspring,

genistein supplementation increased expression of *Cd8* (P<0.001) and decreased expression of *Foxp3* (P=0.006), *Tgfβ1* (P<0.001), *Pd1* (P=0.021), *Ctla4* (P=0.024) and *Il-6* (P=0.014) in responding tumors. In resistant tumors, genistein increased expression of *Il-6* (P=0.037) among HFD offspring. Differences according to t test when comparing the effects of Maternal exposure only and Two-Way ANOVA followed by Holm-Sidak test when comparing the effects of Maternal exposure and offspring treatment. Means ± SEM, n=2-11 are shown.

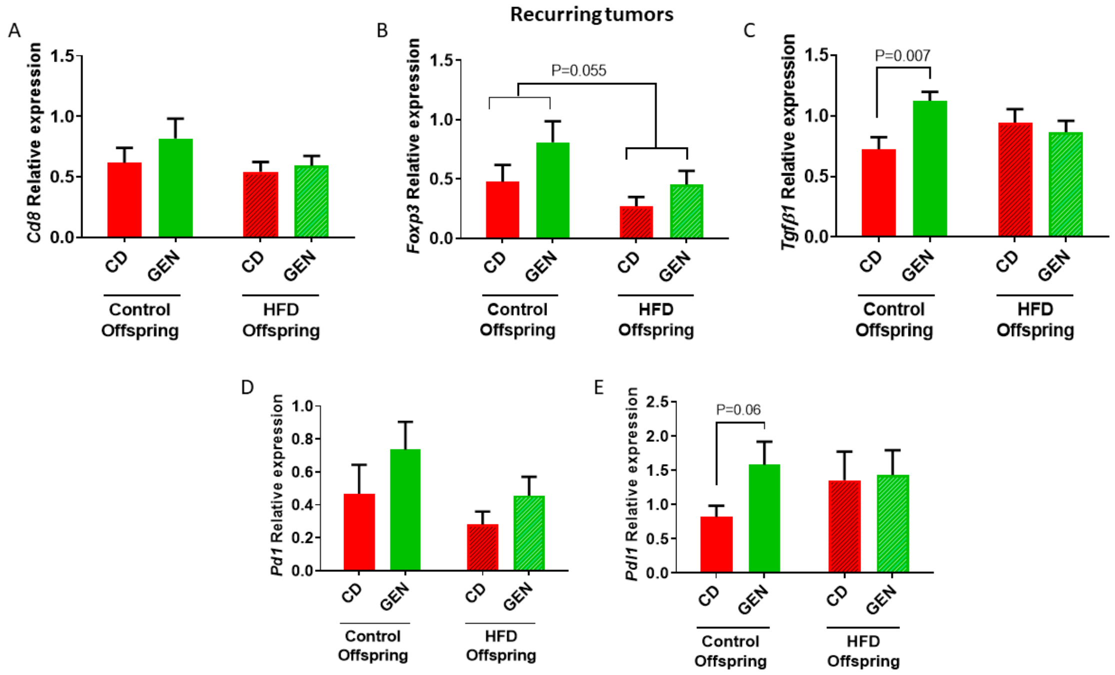

Supplementary Fig. S3. Effects of genistein supplementation on recurring tumors. Gene expression of *Cd8a* (A), *Foxp3* (B), *Tgfβ1* (C), *Pd1* (D) and *Pdl1* (E) in recurring mammary tumors of control and HFD offspring treated with tamoxifen and supplemented with genistein after tamoxifen response. Genistein supplementation significantly and non-significantly increased expression of *Tgfβ1* (P=0.007) and *Pdl1* (P=0.06), respectively, in the control offspring compared to untreated control. Genistein did not change expression of *Cd8*, *Foxp3* and *Pd1* in both control and HFD offspring and *Tgfb1* and *Pdl1* in HFD offspring. Differences according to Two-Way ANOVA followed by Holm-Sidak test for *Foxp3* and *Tgfb1* and t test for *Pdl1*. Means ± SEM, n=7-9 are shown.

Supplementary Table S1. Primers used in quantitative real-time PCR

Gene	Sequence
*Cd8*_ Forward	GCGATATTTACATCTGGGCACC
*Cd8*_ Reverse	AATTTCTCTGAAGGTCTGGGC
*Ctla4*_ Forward	GTACAAAACTCACCTGCAGC
*Ctla4*_ Reverse	AAGGTTGGGTCACTTGTATGG
*Foxp3*_ Forward	TGCCACCTGGGATCAATGTG
*Foxp3*_ Reverse	CGTGGGAAGGTGCAGAGTAGAGC

*Hprt1*_ Forward	5′-GCC CTT GAC TAT AAT GAG CAC T-3′
*Hprt1*_ Reverse	5′-CCG CTG TCT TTT AGG CTT TG-3′
*Il6*_ Forward	TTGCCTTCTTGGGACTGATG
*Il6*_ Reverse	GTGGTATCCTCTGTGAAGTCTC
*Pd1*_ Forward	ATATCCCAGACCCTCACCCAA
*Pd1*_ Reverse	TTCTCTGGCCTCTGACATACCT
*Pdl1*_ Forward	AACGTGACCAGCGTTCTGAG
*Pdl1*_ Reverse	GGTACAGGCAGTTCTGGGATG
*Tgfβ1*_forward	5′- CCTGAGTGGCTGTCTTTTGA -3′
*Tgfβ1*_reverse	5′- CGTGGAGTACATTATCTTTGCTG -3′

Supplementary Table S2. Metabolites significantly dysregulated in gut contents of the offspring of dams fed a high-fat diet (HFD). *N* = 20 per group. CD: offspring of the dams fed a control or low-fat diet.

HMDB ID	Compound_name	*P* value	Fold (HFD/CD)	ESI	RT [min]	Monoisotopic_ Mass	Delta (ppm)
HMDB0000491	3-Methyl-2-oxovaleric acid	8.36E-04	0.40	negative	3.018	130.06	16
HMDB0006528	Docosapentaenoic acid (22n-3)	1.16E-03	0.56	positive	11.502	330.26	0
HMDB0000254	Succinic acid	1.29E-03	2.83	negative	1.184	118.03	13
HMDB0028854	Glycylvaline	3.89E-03	0.70	negative	1.238	174.10	11
HMDB0028735	Asparaginyl-Leucine	5.13E-03	1.81	positive	1.738	245.14	1
HMDB0010378	5,8,11-Eicosatrienoic acid	5.13E-03	0.67	positive	11.806	306.26	0
HMDB0240261	LysoPI(18:0/0:0)	6.15E-03	2.48	negative	9.859	600.33	4
HMDB0000756	Hexanoylcarnitine	6.15E-03	1.44	positive	4.146	259.18	2
HMDB0000156	L-Malic acid	1.12E-02	1.81	negative	0.915	134.02	14
HMDB0000210	Pantothenic acid	1.22E-02	0.72	negative	1.998	219.11	8
HMDB0032549	N-Undecylbenzenesulfonic acid	1.32E-02	1.84	negative	8.245	312.18	1
HMDB0059915	4-Dodecylbenzenesulfonic Acid	1.32E-02	1.78	negative	8.862	326.19	0
HMDB0094651	Pyroglutamylvaline	1.32E-02	1.48	positive	1.826	228.11	0
HMDB0000687	L-Leucine	1.32E-02	0.74	negative	1.316	131.09	15
HMDB0062121	Dihydroferulic acid	1.43E-02	1.65	positive	4.357	196.07	2
HMDB0041607	2-Phenoxyethanol	1.55E-02	1.55	positive	3.611	138.07	2
HMDB0094646	2-Pentanamido-3-phenylpropanoic acid	1.95E-02	0.42	positive	3.851	249.14	2

HMDB0002658	6-Hydroxynicotinic acid	1.95E-02	1.65	negative	2.832	139.03	14
HMDB0061660	2(R)-hydroxydocosanoic acid	2.11E-02	1.94	negative	12.79	356.33	1
HMDB0000867	Ribonic acid	2.11E-02	0.75	negative	1.425	166.05	4
HMDB0000182	L-Lysine	2.11E-02	0.59	positive	1.323	146.11	2
HMDB0000070	Pipecolic acid	2.27E-02	0.70	positive	1.328	129.08	2
HMDB0031491	2,3-Hexanedione	2.45E-02	0.52	positive	3.254	114.07	4
HMDB0001448	Sulfate	2.63E-02	1.92	negative	1.063	97.97	21
HMDB0000300	Uracil	2.63E-02	0.77	negative	0.971	112.03	19
HMDB0028932	Leucyl-Isoleucine	2.63E-02	1.41	positive	3.636	244.18	1
HMDB0002024	Imidazoleacetic acid	2.83E-02	0.76	negative	1.252	126.04	16
HMDB0060390	4-Hydroxyphenyl acetate	2.83E-02	0.78	negative	2.639	152.05	13
HMDB0028991	Phenylalanylaspartic acid	2.83E-02	1.51	positive	3.416	280.11	1
HMDB0037293	2-Acetylpyrrolidine	2.83E-02	1.36	positive	1.444	113.08	3
HMDB0000549	gamma-Butyrolactone	3.04E-02	1.83	positive	1.322	86.04	7
HMDB0000176	Maleic acid	3.04E-02	1.48	negative	0.941	116.01	18
HMDB0001999	Eicosapentaenoic acid	3.26E-02	1.17	positive	3.667	302.22	11
HMDB0000824	propionylcarnitine	3.50E-02	1.21	positive	3.363	217.13	1
HMDB0000138	Glycocholic acid	3.75E-02	1.66	positive	5.873	465.31	1
HMDB0011757	N-Acetylvaline	3.75E-02	1.70	positive	1.818	159.09	1
HMDB0011732	2-Keto-L-gluconate	3.75E-02	1.67	negative	0.897	194.04	9
HMDB0028757	Aspartyl-Leucine	3.75E-02	0.59	negative	2.708	246.12	5
HMDB0028804	Glutaminylphenylalanine	4.02E-02	1.61	positive	3.338	293.14	1
HMDB0001406	Niacinamide	4.02E-02	2.13	positive	1.693	122.05	3
HMDB0031654	3-Aminobutanoic acid	4.30E-02	1.79	positive	1.323	103.06	6
HMDB0029638	4-Methylbenzaldehyde	4.60E-02	0.07	positive	1.647	120.06	3
HMDB0031598	2,3-Pentanedione	4.60E-02	0.49	positive	1.351	100.05	4
HMDB0094704	N-Propionylmethionine	4.60E-02	0.69	positive	3.887	205.08	2
HMDB0032616	Sinapic acid	4.60E-02	1.92	positive	4.245	224.07	1
HMDB0001186	N1-Acetylspermine	4.60E-02	0.40	positive	1.238	244.23	1
HMDB0001227	Thymidine 5'-monophosphate	4.60E-02	2.02	negative	1.128	322.06	4
HMDB0094701	N-Acetylproline	4.91E-02	0.71	positive	3.882	157.07	3
HMDB0001432	Agmatine	4.91E-02	2.65	positive	1.232	130.12	2
HMDB0000562	Creatinine	4.91E-02	1.52	positive	1.332	113.06	4
HMDB0000301	Urocanic acid	4.91E-02	0.73	negative	0.901	138.04	14

Supplementary Table S3. The Lilikoi algorithm identified biological pathways significantly enriched in the fecal samples in the offspring of dams maternal high-fat diet or with or without genistein supplementation. HFD: The offspring of dams fed a maternal high-fat diet. CD: the offspring of dams fed a maternal control diet. CD/GEN: rats from control dams with the genistein supplementation. CD/CD: rats from control dams without the genistein supplementation. HFD/GEN: rats from HFD dams with the genistein supplementation. HFD/CD: rats from HFD dams without the genistein supplementation. GEN: with genistein supplementation. CD: without genistein supplementation.

Pathway	Gain Ratio	Enriched
Warburg Effect	0.42	HFD vs CD
Arginine and Proline Metabolism	0.42	GEN vs CD
Arginine: Glycine Amidinotransferase Deficiency (AGAT Deficiency)	0.42	GEN vs CD
Creatine deficiency, guanidinoacetate methyltransferase deficiency	0.42	GEN vs CD
Guanidinoacetate Methyltransferase Deficiency (GAMT Deficiency)	0.42	GEN vs CD
Hyperornithinemia with gyrate atrophy (HOGA)	0.42	GEN vs CD
Hyperornithinemia-hyperammonemia-homocitrullinuria [HHH-syndrome]	0.42	GEN vs CD
Hyperprolinemia Type I	0.42	GEN vs CD
Hyperprolinemia Type II	0.42	GEN vs CD
L-arginine:glycine amidinotransferase deficiency	0.42	GEN vs CD
Ornithine Aminotransferase Deficiency (OAT Deficiency)	0.42	GEN vs CD
Prolidase Deficiency (PD)	0.42	GEN vs CD
Prolinemia Type II	0.42	GEN vs CD
Disulfiram Action Pathway	0.57	HFD/GEN vs HFD/CD
Nicotinate and Nicotinamide Metabolism	0.57	HFD/GEN vs HFD/CD
3-Phosphoglycerate dehydrogenase deficiency	0.47	HFD/GEN vs HFD/CD
Ammonia Recycling	0.47	HFD/GEN vs HFD/CD
Carnitine Synthesis	0.47	HFD/GEN vs HFD/CD
Dihydropyrimidine Dehydrogenase Deficiency (DHPD)	0.47	HFD/GEN vs HFD/CD
Dimethylglycine Dehydrogenase Deficiency	0.47	HFD/GEN vs HFD/CD
Glycine and Serine Metabolism	0.47	HFD/GEN vs HFD/CD
Hyperglycinemia, non-ketotic	0.47	HFD/GEN vs HFD/CD
Ibuprofen Action Pathway	0.47	HFD/GEN vs HFD/CD
Non Ketotic Hyperglycinemia	0.47	HFD/GEN vs HFD/CD
Sarcosinemia	0.47	HFD/GEN vs HFD/CD
Amino Sugar Metabolism	0.46	HFD/GEN vs HFD/CD
G(M2)-Gangliosidosis: Variant B, Tay-sachs disease	0.46	HFD/GEN vs HFD/CD
Salla Disease/Infantile Sialic Acid Storage Disease	0.46	HFD/GEN vs HFD/CD
Sialuria or French Type Sialuria	0.46	HFD/GEN vs HFD/CD
Tay-Sachs Disease	0.46	HFD/GEN vs HFD/CD
2-ketoglutarate dehydrogenase complex deficiency	0.57	CD/GEN vs CD/CD
Citric Acid Cycle	0.57	CD/GEN vs CD/CD
Congenital lactic acidosis	0.57	CD/GEN vs CD/CD
Fumarase deficiency	0.57	CD/GEN vs CD/CD
Mitochondrial complex II deficiency	0.57	CD/GEN vs CD/CD
Pyruvate dehydrogenase deficiency (E2)	0.57	CD/GEN vs CD/CD
Pyruvate dehydrogenase deficiency (E3)	0.57	CD/GEN vs CD/CD
Methylenetetrahydrofolate Reductase Deficiency (MTHFRD)	0.47	CD/GEN vs CD/CD
Tryptophan Metabolism	0.47	CD/GEN vs CD/CD
Carnitine Synthesis	0.46	CD/GEN vs CD/CD

Supplementary Table S4. Top 20 important metabolites based on mean decrease accuracy identified by the Random Forest algorithms contributing to the classification of the genistein supplementation status.

HMDB ID	ESI	Compound	MeanDecreaseAccuracy	MeanDecreaseGini
HMDB0000884	negative	Ribothymidine	6.87	1.27
HMDB0003306	negative	Phloretin	5.92	1.02
HMDB0000875	positive	Trigonelline	4.83	0.43
HMDB0011500	negative	LysoPE(14:0/0:0)	4.82	0.54
HMDB0004157	positive	Stercobilinogen	4.36	0.48
HMDB0004157	negative	Stercobilinogen	3.98	0.45
HMDB0000054	positive	Bilirubin	3.69	0.40
HMDB0000257	negative	Thiosulfate	3.63	0.38
HMDB0000054	negative	Bilirubin	3.60	0.33
HMDB0000151	positive	Estradiol	3.59	0.35
HMDB0004284	negative	Tyrosol	3.26	0.29
HMDB0002670	negative	Naringenin	2.70	0.35
HMDB0000626	negative	Deoxycholic acid	2.33	0.14
HMDB0002639	positive	Sulfolithocholylglycine	2.32	0.10
HMDB0035248	positive	2,6-Dimethylpyrazine	2.30	0.19
HMDB0000017	negative	4-Pyridoxic acid	2.28	0.18
HMDB0005199	positive	(R)-Salsolinol	2.28	0.27
HMDB0011177	positive	Phenylalanylproline	2.16	0.15
HMDB0000254	negative	Succinic acid	2.07	0.10
HMDB0034423	negative	2-Deoxycastasterone	1.99	0.13
